# TCF7L1 indicates prognosis and promotes proliferation through activation of Keap1/NRF2 in gastric cancer

**DOI:** 10.1093/abbs/gmz015

**Published:** 2019-02-28

**Authors:** Beili Zhang, Jugang Wu, Yantao Cai, Meng Luo, Bing Wang, Yan Gu

**Affiliations:** Department of General Surgery, Shanghai Ninth People’s Hospital, Shanghai Jiao Tong University School of Medicine, Shanghai, China

**Keywords:** gastric cancer, TCF7L1, antioxidant response, Keap1, NRF2

## Abstract

Gastric cancer is one of the most common cancers worldwide and is the third leading cause of cancer-related deaths globally. Although significant progress has been made in the diagnosis and treatment for the cancer, less improvement has been made in overall survival rate. Thus, there is an urgent need for a better understanding of the biological aspects of the cancer. The transcription factor transcription factor 7-like 1 (TCF7L1) is an embryonic stem cell signature gene that is upregulated in multiple aggressive cancer types, but its role in gastric cancer has seldom been discussed. In the present study, by using the Cancer Genome Atlas dataset analysis, we demonstrated that patients with higher expression of TCF7L1 could be used to reflect prognosis. An examination of the mechanisms demonstrated that TCF7L1 could positively regulate antioxidant response in gastric cancer cells by positively regulating Keap1/NRF2 [Kelch-like ECH-associated protein 1/nuclear factor (erythroid-derived 2)-like 2] pathway. Collectively, our data demonstrated that TCF7L1 is a novel marker for predicting overall survival of gastric cancer and provided the possible underlying molecular mechanism.

## Introduction

Gastric cancer is one of the leading causes of mortality from neoplastic disease and is particularly prevalent in Asian populations [1,2]. Early detections and diagnosis of gastric cancer through endoscopic methods and subsequent treatment by submucosal resection has achieved a 5-year survival rate of up to 95% in cohort studies. However, the overall 5-year survival of all patients with gastric cancer is only ~26%, as many of the patients have regional or distant metastasis at the time of diagnosis [3,4]. Thus, there is an urgent need for improved methods to facilitate the early diagnosis of cancer and pre-malignant lesions of the stomach. Furthermore, an understanding of the molecular biological aspects of the cancer is more vital than ever to ensure early and effective treatment [5].

Cancer cells share many similar characteristics in common with stem cells. Previous studies demonstrated that by comparing the expression profiles of signature genes in several types of poorly differentiated and aggressive tumors to those of embryonic stem (ES) cells, a subset of ES-specific transcriptional regulators was highly enriched. One such transcriptional regulator was transcription factor transcription factor 7-like 1 (TCF7L1, also known as TCF3) [6–8]. TCF7L1 is a member of the lymphoid enhancer factor/T cell factor (LEF/TCF) family of transcription factors, which interact with the Wnt signaling pathway regulator, β-catenin and act as DNA-specific binding proteins. TCF7L1 contains a highly conserved DNA binding high-mobility group-box and a conserved β-catenin binding domain at the amino terminus [9]. Through interacting with β-catenin, TCF7L1 regulates the expressions of a series of Wnt target genes [10]. The expression status of TCF7L1 has been found to be upregulated in high-grade tumors and is associated with poor survival. In breast cancer, for example, higher expression of TCF7L1 indicates worse prognosis, and silencing of TCF7L1 expression decreases tumor growth and metastasis. In colorectal cancer, TCF7L1 positively regulates cell proliferation, and silencing its expression reduces the size of xenografted tumors [11]. The implications of TCF7L1 in breast cancer and colorectal cancer have been attributed to its regulatory roles in the Wnt/β-catenin pathway [12]. In gastric cancer, the canonical Wnt signaling pathway plays crucial roles in regulating proliferation, stem cell maintenance, and homeostasis in normal gastric mucosa. Furthermore, it is recognized that dysregulation of the Wnt pathway plays a critical role in gastric cancer development, with more than 30% of gastric cancer in which activated Wnt/β-catenin signaling can be found. Several components of the Wnt/β-catenin pathway may be overexpressed or their function was increased by diverse mechanism [13,14]. What is more, the loss of Wnt/β-catenin pathway inhibitors also play decisive roles in gastric cancer carcinogenesis, progression, and has clinical implications [15]. As an important regulator of Wnt/β-catenin pathway, TCF7L1 is implicated in gastric cancer prognosis, however the underlying molecular mechanism of TCF7L1 in gastric cancer has seldom been discussed.

In the present study, we explored the roles of TCF7L1 in gastric cancer prognosis by using Cancer Genome Atlas (TCGA) dataset analysis. *In vitro* cell line studies demonstrated that TCF7L1 negatively regulated Keap1 expression, leading to constitutive activation of NRF2 and NRF2-mediated glycolysis and antioxidant-response. Our finding established an important role for TCF7L1 in gastric cancer proliferation and shed light on the mechanisms underlying its tumorigenic function.

## Materials and Methods

### Cell culture

Human gastric cancer cell lines AGS and MGC-803 were obtained from Cell Bank of Institute of Biochemistry and Cell Biology, the Chinese Academy of Sciences (Shanghai, China). The two cell lines were grown in Dulbecco’s modified Eagle medium (Invitrogen, Carlsbad, USA) supplemented with 10% fetal bovine serum and 1% penicillin–streptomycin at 37°C with humidified 5% CO_2_.

### Establishment of TCF7L1 knockdown cell lines

The lentivirus-mediated transfection method was used to generate stable TCF7L1 knockdown gastric cancer cell lines. Next, the pLKO.1-TRC cloning vector obtained from Addgene (Watertown, USA) was used to express shRNA oligoes against TCF7L1 [16]. The two 21-bp shRNAs targeting against TCF7L1 were 5′-ATCCGAGCTGTCACCGTATTA-3′ and 5′-TCCAGCACACTTGTCTAATAA-3′, respectively. Scramble shRNA or Scr (Addgene plasmid 1864; Addgene, Watertown, USA) was used as negative controls. Lentiviral particles were produced by co-transfecting TCF7L1 silencing constructs with psPAX2 and pMD2.G vectors in a ratio of 4:3:1 into HEK293T cells. Stable TCF7L1 knockdown cell lines were obtained by infecting AGS and MGC-803 cells with lentiviral particles and subsequent puromycin selection.

### Cell viability assay

Cell Counting kit-8 (Dojindo, Tokyo, Japan) was used to measure cell viability. Briefly, 200 μl of medium containing cells (3000/well) was seeded into 96-well plates. After culturing for indicated time, CCK-8 solution was added into each well at 37˚C. After 2 h of incubation, the optical density values of each well were measured using a microplate reader (BioTek, Winooski, USA) at 450 nm.

### Colony-formation assay

AGS and MGC-803 cells (5 × 10^2^) stably expressing shRNA targeting against TCF7L1 and the relative control cells were seeded. After cultivating for 10 days, 4% paraformaldehyde was used to fix the cells, followed by staining with 1% crystal violet. The colonies were counted subsequently under a microscope.

### Quantitative real-time PCR

Total RNA was extracted by using Trizol reagent (Invitrogen). TaKaRa PrimeScript RT reagent kit (Dalian, China) was used for reverse transcription to obtain cDNA. The expressions of candidate genes and β-actin were determined by quantitative real-time PCR with an ABI 7900HT Real-Time PCR system (Applied Biosystems, Foster City, USA). Primer sequences of *TCF7L1*, heme oxygenase 1 (*HMOX1*), malic enzyme 1 (*ME1*), *Keap1*, *NRF2*, glutamate–cysteine ligase catalytic subunit (*GCLC*), glutamate–cysteine ligase modifier subunit (*GCLM*), thioredoxin reductase 1 (*TXNRD1*), *NADP(H)* quinone dehydrogenase 1 (*NQO1*), and *β*-*actin* are listed in **Table 1** [17].

**Table 1.** Sequence of primers used in this study

Gene	Sequence	
*TCF7L1*	Forward	5′-TGCAGCCTCCTCCTCTGGGCAGAT-3′
	Reverse	5′-GGGGGAGCTTAGTGGGCAGACTTG-3′
*HMOX1*	Forward	5′-AGCGGGCCAGCAACAAAGTGCAA-3′
	Reverse	5′-CAGCATGCCTGCATTCACATGGC-3′
*ME1*	Forward	5′-CCTCACTACTGCTGAGGTTATAGC-3′
	Reverse	5′-CGGTTCAGGATAAACTGTGGCTG-3′
*Keap1*	Forward	5′-CACCACAACAGTGTGGAGAGGTA-3′
	Reverse	5′-TACAGTTGTGCAGGACGCAGACG-3′
*NRF2*	Forward	5′-CCAATTCAGCCAGCCCAGCACAT-3′
	Reverse	5′-CCAATTCAGCCAGCCCAGCACAT-3′
*GCLC*	Forward	5′-GTGGTACTGCTCACCAGAGTG-3′
	Reverse	5′-AGCTCCGTGCTGTTCTGGGCCTT-3′
*GCLM*	Forward	5′-ATCTTGCCTCCTGCTGTGTGATGC-3′
	Reverse	5′-CAATGACCGAATACCGCAGTAGCC-3′
*TXNRD1*	Forward	5′-GCAATCCAGGCAGGAAGATTGCT-3′
	Reverse	5′-CTCTTGACGGAATCGTCCATTCC-3′
*NQO1*	Forward	5′-CGGAGTAAGAAGGCAGTGCTTTC-3′
	Reverse	5′-TCTGCTGGAGTGTGCCCAATGCT-3′
*β-actin*	Forward	5′-CCAACCGCGAGAAGATGACCCA-3′
	Reverse	5′-ATCACGATGCCAGTGGTACG-3′

### Western blot analysis

Cells were washed with ice-cold phosphate-buffered saline (PBS) and lysed in ice-cold RIPA buffer (150 mM NaCl, 1% NP-40, 50 mM Tris/HCl, pH 8.0, and 10% glycerol) containing protease and phosphatase inhibitors purchased from Selleck (Shanghai, China). Cell debris was removed by centrifugation at 10000 *g* for 20 min at 4°C. Protein concentrations of the whole cell lysate were measured by using BCA Protein Assay kit (Thermo Pierce, Rockford, USA). Equal amount of total proteins were subject to SDS-PAGE separation and then transferred to PVDF membranes (Millipore, Billerica, USA). Then, the membranes were incubate in 1 × TBST (Tris-buffered saline with Tween-20) with 5% (*w*/*v*) non-fat dry milk. Subsequently, the membrane was incubated with indicated primary antibodies followed by washing with TBST and incubation with secondary antibody that conjugated with Horseradish Peroxidase (HRP). Omin-ECL FemtoLight Chemiluminescence kit (EpiZyme, Shanghai, China) was used to detect the western blot HRP-substrate signals. Antibodies against TCF7L1 and NRF2 were purchased from Abcam (Cambridge, UK). Antibodies against Keap1 β-actin and HRP-conjugated secondary antibody were obtained from Proteintech Group (Rosemont, USA).

### Flow cytometry analysis of ROS generation and intracellular GSH activity assay

Intracellular reactive oxygen species (ROS) was detected using an oxidant-sensitive fluorescent probe (DCFH-DA, Beyotime, Nantong, China). In brief, cells were washed twice with PBS. Then, cells were stained with 10 μM DCFH-DA at 37°C for 20 min according to the manufacturer’s instruction. DCFH-DA was deacetylated intracellularly by non-specific esterase, which was further oxidized by ROS to the fluorescent compound 2,7-dichlorofluorescein (DCF). DCF fluorescence was detected with a FACScan flow cytometer (Becton Dickinson, Franklin Lakes, USA). To determine the oxidative status, intracellular GSH activity was measured using the GSH/GSSG Ratio Detection Assay kit (Abcam).

### Extracellular acidification rate and oxygen consumption rate

Cellular glycolytic capacity and mitochondrial function were measured with the Seahorse Bioscience XF96 Extracellular Flux Analyzer (Santa Clara, USA) using the Seahorse XF Glycolysis Stress Test kit (Agilent, Santa Clara, USA) and Cell Mito Stress Test kit (Agilent), respectively, according to the manufacturer’s instructions.

### Analysis of promoter activity with dual-luciferase assay

Cells were plated on 96-well culture plates and transfected using Lipofectamine™ 2000 (Invitrogen). Gastric cancer cells were transfected with the Keap1 promoter construct (pGL3-Keap1) or pFMARE-luc construct with the *Renilla* luciferase expression vector, pRL-TK (Promega, Madison, USA). pFMARE-luc construct was obtained from Genomeditech (Shanghai, China). Next, the cells were assayed for both firefly and *Renilla* luciferase activities using a dual-luciferase system (Promega, Madison, USA) as described in the manufacturer’s protocols.

### Chromatin-immunoprecipitation assay

The coding sequence of TCF7L1 was constructed into pCMV-C-FLAG vector (Beyotime, Nantong, China) to generate FLAG-tagged TCF7L1 expressing constructs. pCMV–TCF7L1–FLAG was transfected into gastric cancer cells. ChIP was performed according to the instructions of the Magna ChIP™ A/G Chromatin-Immunoprecipitation kit (Millipore). Anti-FLAG antibody (Sigma, St Louis, USA) was used to perform ChIP assay. The nuclear DNA extracts were amplified using a pair of primers that spanned the Kerp1 promoter region. The sequences were: 5′-GGCGCTGTGCGTTGTTAAAAGGAGA-3′ (forward) and 5′-AGGTCGCCTCCGTAGGGGGTCCCTC-3′ (reverse) [17]. As a negative control, the primary antibody was omitted or replaced with normal mouse immunoglobulin G.

### TCGA dataset analysis

TCGA-STAD on RNA expression (Level 3) of gastric cancer patients in terms of RNA-seq by Expectation–Maximization was downloaded from the Cancer Genomics Brower of the University of California, Santa Cruz (https://genome-cancer.ucsc.edu/). In total, 360 primary gastric cancer samples from patients with detailed expression data were chosen from the updated TCGA database according to parameters mentioned.

### Statistical analysis

Statistical analyses were performed with the SPSS software (version 17.0, IBM Corp., Armonk, USA) using independent *t*-tests (for continuous variables) and Pearson’s *χ*^2^-tests (for categorical variables). Logistic regression was used to determine the correlation between TCF7L1 and Keap1 expression level and clinicopathological characteristics in the TCGA cohorts. Statistical significance was based on two-sided *P* values of <0.05.

## Results

### TCF7L1 expression predicts prognosis of gastric cancer patients

**Figure 1.**
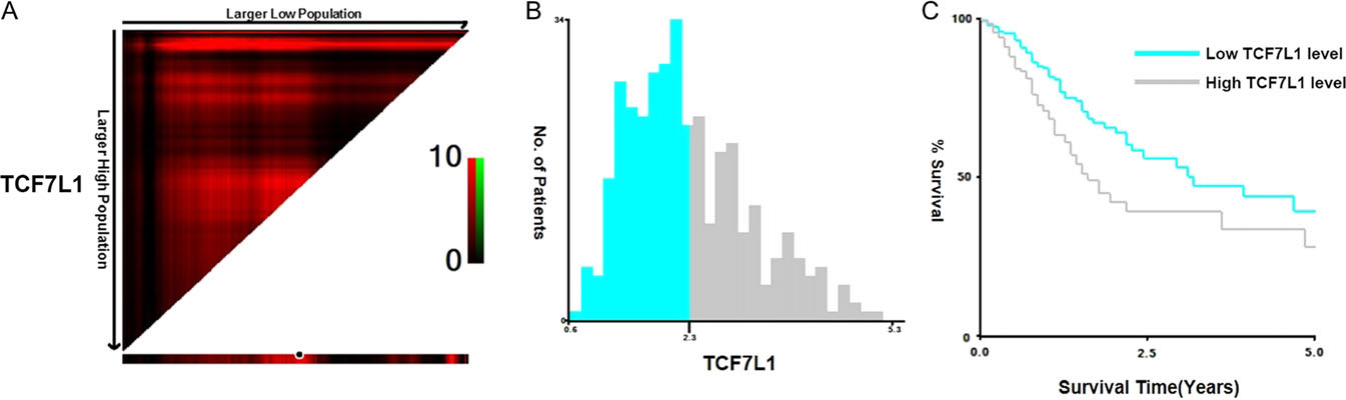
**TCF7L1 expression predicts prognosis of gastric cancer patients** X-tile analysis of 5-year OS was performed using patients’ data in TCGA database to determine the optimal cut-off value TCF7L1 expression. The sample of gastric cancer patients was equally divided into training and validation sets. (A) X-tile plots of training sets are shown in the left panels, with plots of matched validation sets shown in the smaller inset. (B) The optimal cut-off values highlighted by the black circles in left panels are shown in histograms of the entire cohort. The optimal cut-off value for TCF7L1 was 2.3. (C) Kaplan–Meier plots are displayed. *P* values were determined by using the cut-off values defined in training sets and applying them to validation sets. Results indicated that patients with higher levels of TCF7L1 displayed shorter survival (*P* = 0.001).

To investigate the impact of TCF7L1 expression on gastric cancer malignancy, we used the survival analysis for high and low TCF7L1 expressions in gastric cancer patients from TCGA database. Higher TCF7L1 expression was shown to predict poorer survival compared with lower TCF7L1 expression in gastric cancer patients (**Fig. 1A,C**). Next, the clinicopathological parameters of the TCGA-derived gastric patients were analyzed, and results demonstrated that patients with higher TCF7L1 expression exhibited older age, advanced T and N stages (**Table 2**).

**Table 2.** Clinical characteristics of patients with gastric in TCGA

Variable	Keap1		*P*	TCF7L1		*P*
	Low	High		Low	High	
	(*n* = 135)	(*n* = 225)		(*n* = 210)	(*n* = 150)
Sex			0.864			0.218
Male	87	147		142	92	
Female	48	78		68	58	
Age			0.071			0.001
≤60	54	69		57	66	
>60	81	156		153	84	
Grade			0.379			0.069
G1/G2	44	89		88	45	
G3	88	130		117	101	
Gx	3	6		5	4	
T stage			0.014			0.002
T1/2	23	64		63	24	
T3/4	112	161		147	126	
N stage			0.084			0.011
Negative	35	78		77	36	
positive	100	147		133	114	
M stage			0.818			0.434
M0	122	206		193	135	
M1	8	10		8	10	
Mx	5	9		9	5	

### TCf7l1 knockdown impairs gastric cancer cell proliferation

**Figure 2.**
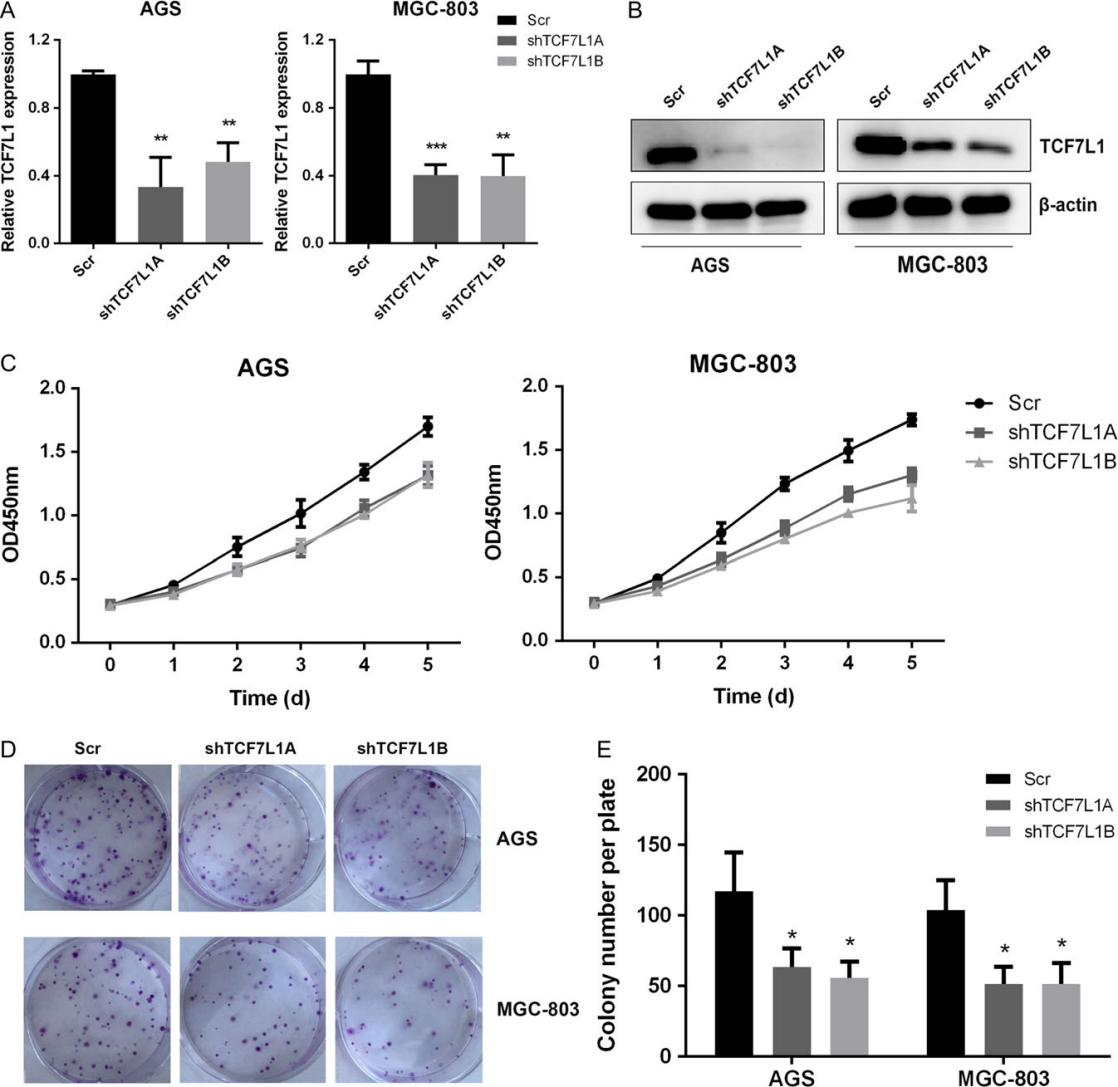
**TCF7L1 knockdown impairs gastric cancer cell proliferation** *In vitro* cell proliferation assay was performed to determine the role of TCF7L1 on gastric cancer cell proliferation. (A) The efficacy of TCF7L1 knockdown was validated by real-time PCR analysis. (B) Western blot analysis using anti-TCF7L1 antibody further validated the knockdown effect. (C) TCF7L1 silencing inhibited cell viability. (D,E) TCF7L1 knockdown impaired colony-formation capacity of AGS and MGC-803 cells.

To assess the impact of TCF7L1 on gastric cancer cell proliferation, we silenced TCF7L1 expression by utilizing lentivirus-mediated transfection method in AGS and MGC-803 cells. The efficiency of silencing was confirmed by quantitative real-time PCR and western blot analysis (**Fig. 2A,B**). Next, we performed CCK-8 cell proliferation assay. As shown in **Fig. 2C**, silencing TCF7L1 expression in AGS and MGC-803 cells significantly inhibited cell proliferation. Then, colony-formation assay was performed to further validate the impact of TCF7L1 on colony-formation capacity in AGS and MGC-803 cells. The results showed that silencing of TCF7L1 expression significantly inhibited colony-formation capacities of AGS and MGC-803 cells (**Fig. 2D,E**).

### TCF7L1 regulates aerobic glycolysis and antioxidant response

**Figure 3.**
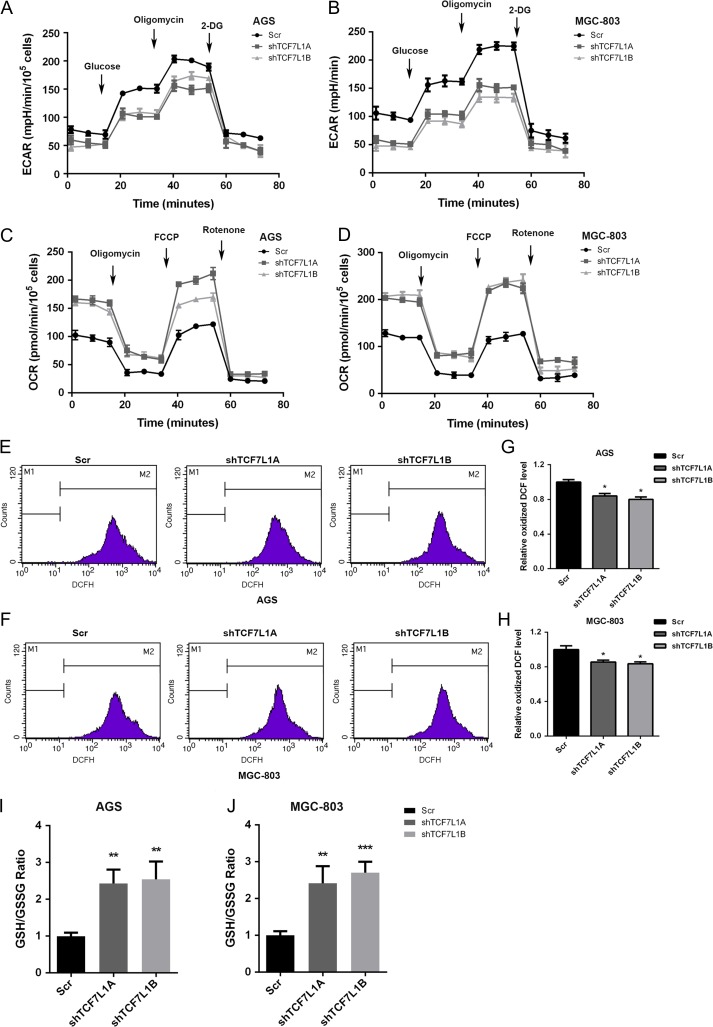
**TCF7L1 regulates aerobic glycolysis and antioxidant response** In order to determine the underlying molecular mechanism that governing cancer cell proliferation by TCF7L1, glycolysis and antioxidant capacity were measured. (A,B) Silencing of TCF7L1 expression inhibited ECAR rates in AGS and MGC-803 cells, indicating that TCF7L1 is a positive regulator of aerobic glycolysis. (C,D) TCF7L1 knockdown increased OCR values, suggesting that TCF7L1 might negatively regulate mitochondrial respiration in AGS and MGC-803 cells. (E–H) As determined by ROS measurement, silencing TCF7L1 decreased intracellular ROS levels. (I,J) To confirm the impact of TCF7L1 on intracellular redox balance maintenance, GSH/GSSG ratio was measured. Silencing of TCF7L1 increased GSH/GSSG ratios in AGS and MGC-803 cells, indicating that TCF7L1 could maintain intracellular redox balance.

To further analyze the physiological functions of TCF7L1 in gastric cancer cells, we first assessed the impact of TCF7L1 on aerobic glycolysis which is a process that provides cancerous cells with energy supply and building blocks for macromolecule synthesis. As shown in **Fig. 3A,B**, silencing TCF7L1 expression significantly inhibited extracellular acidification rate (ECAR) rate in AGS and MGC-803 cells, indicating that TCF7L1 was a positive regulator of glycolysis. Furthermore, oxygen consumption rate (OCR) measurement demonstrated that silencing TCF7L1 increased OCR values, which further reflected that TCF7L1 could positively regulate mitochondrial respiration and functions as a positive regulator of aerobic glycolysis (**Fig. 3C,D**). It is well accepted that ROS production in cancer cells positively regulates aerobic glycolysis. Thus, we assessed the impact of TCF7L1 on ROS generation in AGS and MGC-803 cells. As shown in **Fig. 3E,H**, silencing TCF7L1 expression significantly attenuated the generation of ROS. Finally, we examined the impact of TCF7L1 on redox balance in gastric cancer cells by performing GSH/GSSG ratio analysis. Results showed that silencing TCF7L1 expression in AGS and MGC-803 cells increased GSH/GSSG ratio, indicating that TCF7L1 is a key regulator in maintaining the redox balance in gastric cancer cells (**Fig. 3I,J**).

### TCF7L1 positively regulates NRF2 at protein level and NRF2 downstream transcription program

**Figure 4.**
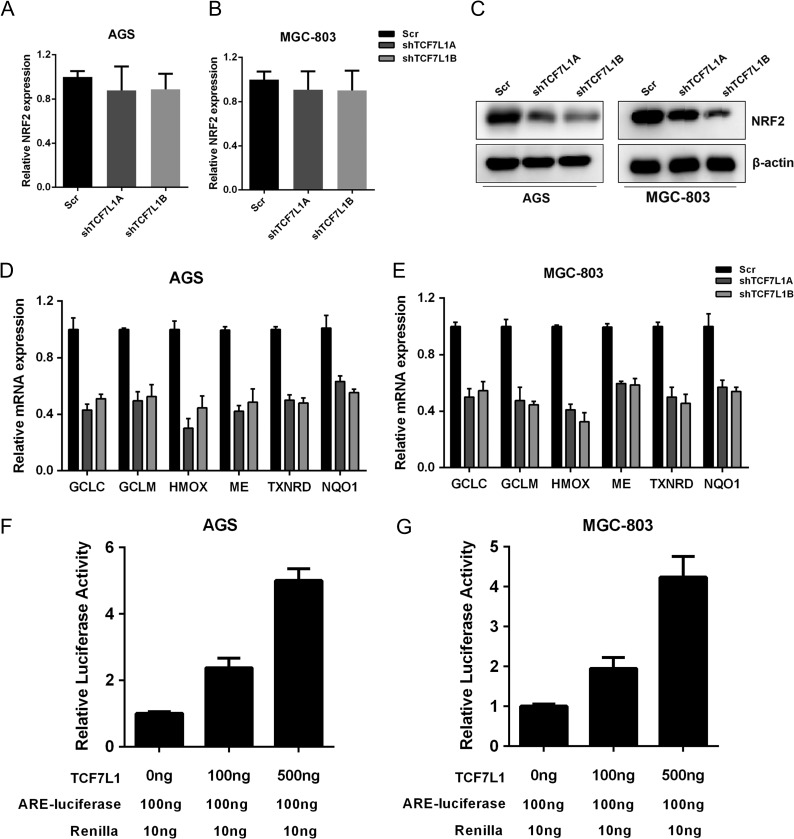
**TCF7L1 positively regulates NRF2 at protein level and NRF2 downstream transcription program** NRF2 is an important regulator of glycolysis and intracellular redox balance, thus we measured the impact of TCF7L1 on NRF2 expression. (A,B) Silencing of TCF7L1 expression had slight impact on NRF2 transcription. (C) NRF2 protein level was decreased in TCF7L1-silenced AGS and MGC-803 cells. (D,E) Silencing of TCF7L1 expression decreased the transcription of NRF2-targeted antioxidant genes, including *GCLC*, *GCLM*, *HMOX*, *ME*, *TXNRD*, and *NQO1*. (F,G) NRF2 transcription activity was measured by ARE-luciferase activity assay. TCF7L1 increased ARE-luciferase activity in a dose-dependent manner.

It is well acknowledged that NRF2 is a positive regulator of aerobic glycolysis, antioxidant response, and redox state maintenance. Thus, we assessed the impact of TCF7L1 on NRF2 expression and its downstream transcription program. First, we examined the impact of TCF7L1 on the transcription of NRF2, and the results showed that silencing TCF7L1 only minimally impacted NRF2 transcription (**Fig. 4A,B**). However, in TCF7L1-silenced AGS and MGC-803 cells, the protein level of NRF2 was significantly decreased, which indicated that TCF7L1 might regulate NRF2 expression at the post-transcription levels (**Fig. 4C**). Next, we measured the NRF2 target genes that might participate in the antioxidant response and ROS detoxification. As shown in **Fig. 4D,E**, NRF2 knockdown significantly decreased the expressions of NRF2 targets including *GCLC*, *GCLM*, *HMOX1*, *ME1*, *TXNRD1*, and *NQO1*. NRF2 transcription activity can be reflected by ARE-luciferase activity, we therefore assessed the impact of TCF7L1 on ARE-luciferase activity. Our results showed that TCF7L1 could positively regulate ARE-luciferase activity in a dose-dependent manner in AGS and MGC-803 cells (**Fig. 4F,G**). Collectively, these data suggested that TCF7L1 could regulate NRF2 at protein level and act as a positive regulator of NRF2 transcription program.

### TCF7L1 negatively regulates Keap1 expression in gastric cancer cells

**Figure 5.**
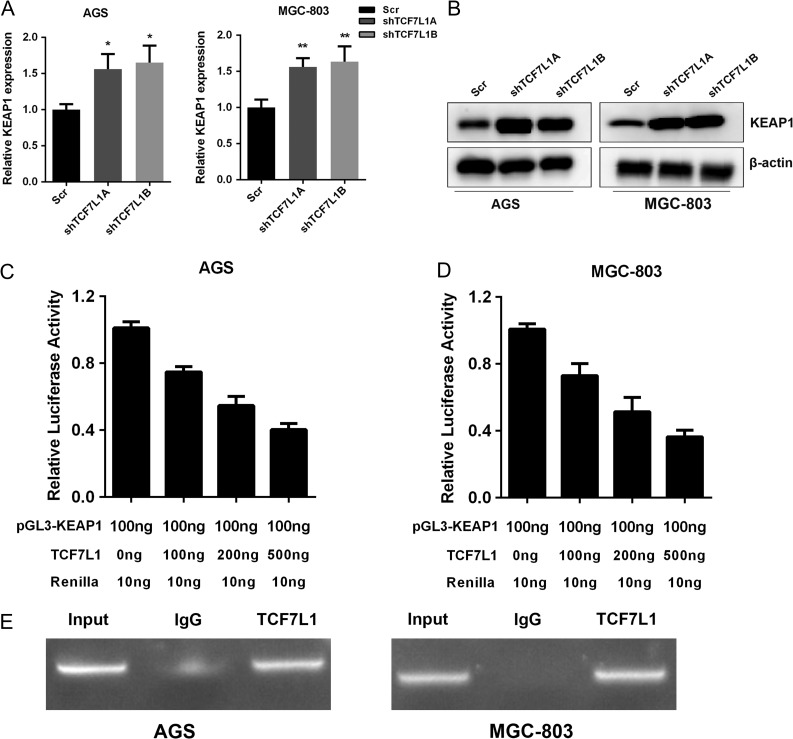
**TCF7L1 negatively regulates Keap1 expression in gastric cancer cells** TCF7L1 could influence NRF2 in protein level. We hypothesized that this might due to the impact of TCF7L1 on Keap1 transcription, which could regulate NRF2 at the post-translational level. (A) Silencing of TCF7L1 increased Keap1 at mRNA level in AGS and MGC-803 cells. (B) Silencing of TCF7L1 expression increased Keap1 at protein level. (C,D) TCF7L1 could inhibit Keap1 promoter activity in a dose-dependent manner. (E) ChIP results validated that TCF7L1 could bind with the Keap1 promoter region.

As shown above, TCF7L1 could regulate NRF2 at protein level. It has been documented that Keap1 functions as a negative regulator of NRF2 protein stability. Thus, we examined the mRNA levels of Keap1 in TCF7L1-silenced AGS and MGC-803 cells. Our results showed that silencing TCF7L1 expression increased Kera1 at both mRNA and protein levels, which suggested that *Kera1* might be a downstream target of TCF7L1 (**Fig. 5A,B**). Next, we cloned the Keap1 promoter into pGL3-Basic vector to examine the impact of TCF7L1 on promoter activity of Keap1. As demonstrated by dual-luciferase assay, TCF7L1 inhibited Keap1 promoter activity in a dose-dependent manner (**Fig. 5C,D**). Finally, we performed ChIP assay to test whether TCF7L1 could bind with the promoter region of Keap1. The ChIP assay results showed that TCF7L1 could occupy the promoter region of Keap1 in gastric cancer cells (**Fig. 5E**).

### Keap1 expression is negatively correlated with TCF7L1 expression and indicates better prognosis

**Figure 6.**
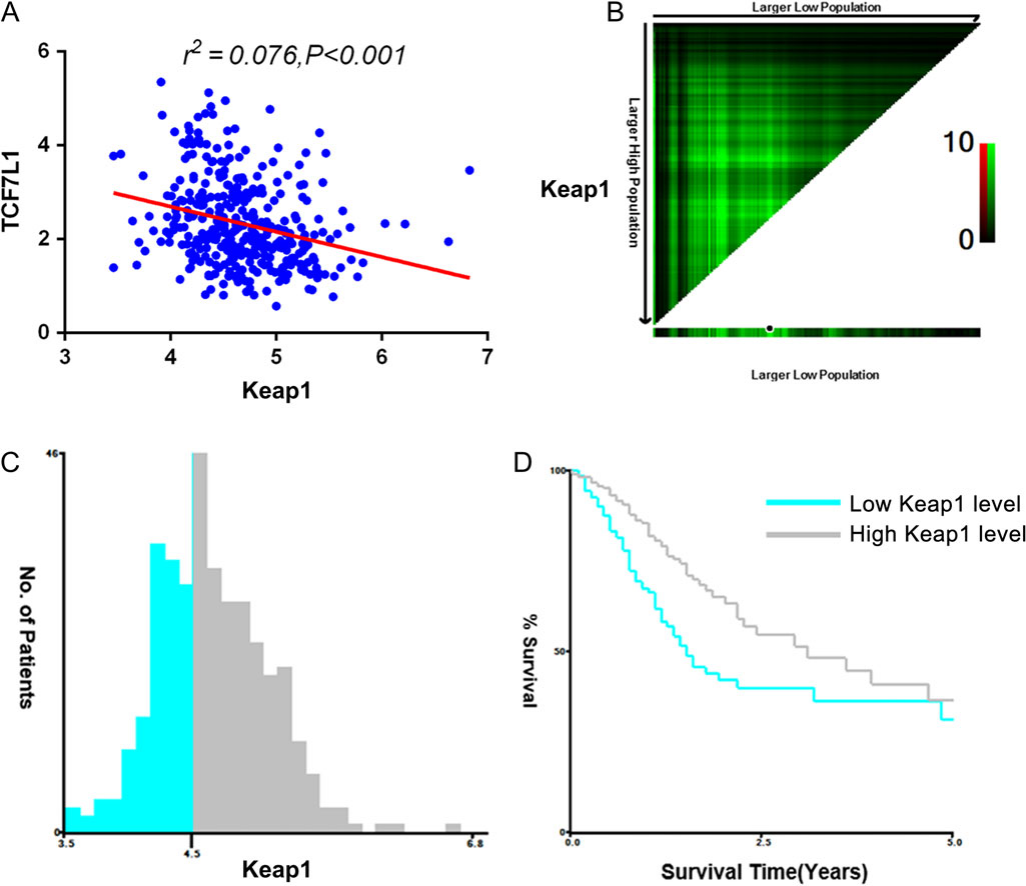
**Keap1 expression is negatively correlated with TCF7L1 expression and indicates better prognosis** To validate the *in vitro* cell line observations, the correlation between TCF7L1 and Keap1 was analyzed in TCGA-included gastric cancer patients. (A) TCF7L1 expression is negatively correlated with Keap1 expression in gastric cancer patients (*r*^*2*^*=*0.076, *P <* 0.001). (B) X-tile plots of training sets are shown in the left panels, with plots of matched validation sets shown in the smaller inset. (C) The optimal cut-off values highlighted by the black circles in left panels are shown in histograms of the entire cohort. The optimal cut-off value for Keap1 was 4.5. (D) Kaplan–Meier plots are displayed. *P* values were determined by using the cut-off values defined in training sets and applying them to validation sets. Results indicated that patients with higher levels of Keap1 displayed better survival (*P* = 0.002).

To verify the *in vitro* observations of the negative correlation between TCF7L1 with Keap1, we performed TCGA dataset analysis of their correlation in gastric cancer patients. As shown in **Fig. 6A**, TCF7L1 was negatively correlated with KEAP1 expression in gastric cancer patients. Next, we assessed the contribution of Keap1 expression to gastric cancer prognosis. It was found that patients with decreased Keap1 expression exhibited a worse prognosis, which was consistent with previous study demonstrating that Keap1 functions as a tumor suppressor (**Fig. 6B,D**). The clinic pathological parameters of TCGA-derived gastric cancer patients were further analyzed and results showed that higher Keap1 expression was associated with advanced T stage (**Table 2**). These results further revealed the impact of TCF7L1 and KEAP1/NRF2 axis in gastric cancer progression.

## Discussion

In the present study, we explored the novel functions of TCF7L1 in gastric cancer prognosis. Our results demonstrated that TCF7L1 promoted gastric cancer cell proliferation by positively regulating aerobic glycolysis and antioxidant response via NRF2, an important player in oncogenesis and progression in many types of cancers. Furthermore, we demonstrated that TCF7L1 could inhibit the expression of Keap1, the negative regulator of NRF2. The implication of Keap1 and its correlation with prognosis of patients with gastric cancer has been observed. These data suggest that elevated expression of TCF7L1 facilitates the proliferation of gastric cancer.

TCF7L1 has been shown to regulate tumor growth in many types of cancers, such as leukemia and colorectal cancer [18,19]. In this study, the same tumor-promoting roles of TCF7L1 were observed in gastric cancer patients. Furthermore, we examined the molecular mechanism from a novel aspect. It is well-accepted that solid tumor cells reside in a microenvironment characterized by decreased oxygen and nutrient supply. To survive under such hostile microenvironment, tumor cells must shift their metabolism pattern to guarantee proliferation [20]. The best characterized metabolism reprogramming is aerobic glycolysis, also known as Warburg effect, which was discovered by Otto Warburg nearly a century ago [21,22]. Through aerobic glycolysis, cancer cells utilized glucose by glycolysis instead of mitochondrial oxidative phosphorylation. However, from the ATP generation aspect, this pattern is ineffective, as only two ATPs are generated from one glucose molecule by aerobic glycolysis, which is far more less than that can be generated by OXPHS. But through aerobic glycolysis, cancer cells break down glucose to generate small molecules for macromolecule synthesis to meet the demand of uncontrolled proliferation [23]. Furthermore, glucose can be transformed into lactate acid, which creates an acidic environment. In acidic environment, the extracellular matrix becomes less stable and easier to be degraded, which facilitates metastasis of cancer cells [24]. Thus, higher expression of TCF7L1 provides metabolic advantage to proliferative gastric cancer cells. These results are consistent with the functions of Wnt/β-catenin in governing metabolism reprogramming in cancers. For example, in hepatocellular carcinoma, the activation of β-catenin has a crosstalk with glycolysis via autophagy-induced expression of monocarboxylate transporter 1, which plays an important role in lactic acid transport and H^+^ clearance in cancer cells [25]. In colon cancer, Wnt/β-catenin drives Warburg metabolism, which directs the use of glucose for cancer cell proliferation and links to vessel delivery of oxygen and nutrients [26]. TCF7L1 could interact with Wnt/β-catenin pathway and the present study is consistent with previous studies that demonstrated the role of Wnt/β-catenin in glycolysis. The observations of TCF7L1 in glycolysis reprogramming might shed light on novel directions for uncovering the roles of TCF7L1 in gastric cancer.

Living cells operate optimally within certain pH and temperature ranges, and they also require optimal redox balance for metabolic processes. Their ability to adapt rapidly to perturbations in homeostasis is essential for their survival. Cancer cells can be threatened by ROS and by toxic secondary metabolites generated from ROS-mediated cell damage, leading to oxidative stress. Constitutive activation of NRF2-ARE protects cancerous cells from oxidative stress [27]. The upregulation of NRF2 has been observed in many types of cancers, such as breast, head and neck, ovarian and pancreatic cancers. The prognosis of patients that exhibit higher level of NRF2 in clinic is usually poor [28–31]. Higher level of NRF2 participates not only in antioxidant response, but also in metabolism reprogramming. For example, in lung cancer cells, NRF2 redirects glucose and glutamine metabolism into anabolic pathways in metabolic reprogramming, which favors uncontrolled proliferation of cancer cells [32]. What is more, NRF2 can also regulate aerobic glycolysis through different mechanisms. For example, in breast cancer, NRF2 regulates aerobic glycolysis via the hypoxia inducible factor 1α [33]. In the present study, we demonstrated that TCF7L1 could influence NRF2 protein level, and the impact of TCF7L1 on antioxidant response and glycolysis might due to its influence on NRF2. Previous studies have demonstrated that the Wnt/β-catenin pathway regulator Axin1 could form a complex with NRF2, and the interaction could regulate antioxidant metabolism in hepatocytes [34]. These findings inspired us to question whether TCF7L1 could interact with NRF2 and act as a co-factor of NRF2 in governing antioxidant response and metabolism reprogramming. Another import role of NRF2 in cancer is that activation of NRF2 is usually linked to chemotherapy and radiotherapy resistance [35]. One critical aspect for poor overall survival of gastric cancer is its innate resistance to chemotherapy [36]. Thus, the present study inspired us to question whether TCF7L1 could play a role in chemotherapy resistance, and whether TCF7L1 could be used as a target to reverse chemotherapy resistance to improve overall gastric cancer survival.

To explore the mechanism that directs TCF7L1 in regulating NRF2, we examined the changes in Keap1 upon TCF7L1 silencing. Keap1 is a substrate adaptor for a Cul3-containing E3 ubiquitin ligase, which regulates NRF2 post-translationally. Our results suggested that TCF7L1 could negatively regulate Keap1 expression. Loss-of-function mutations in Keap1 have been identified in several human cancers [37]. The mutations in the *Keap1* gene were first discovered in common solid tumors [38]. The mutated Keap1 displayed a lower affinity to NRF2 and consequently activated NRF2 in these cells. However, tumors without Keap1 mutations also exhibited constitutively activated NRF2, which might due to decreased Keap1 expression in these cancers. Recently, epigenetic silencing of *Keap*1 gene has been observed in many cancers to promote the accumulation of NRF2. Promoter hypermethylation of *Keap1* has been observed in several types of cancers, such as lung, breast and colorectal cancers [39–41]. However, the expression status of Keap1 in gastric cancer has seldom been discussed in gastric cancer patients. In our present study, we identified *Keap1* as a tumor-suppressor gene which is correlated with better survival of gastric cancer patients. Furthermore, we demonstrated that *Keap1* could be regulated by TCF7L1 which is a transcription factor. However, we did not examine the epigenetic impacts of TCF7L1 on Keap1 expression, which prompted us to perform further investigations to screen TCF7L1-interacting epigenetic partners in gastric cancer. For example, TCF7L1 has been reported to recruit CtBP and HDAC1 to epigenetically repress tumor-suppressor *DICKKOPF4* gene in colorectal cancer cells [18]. Thus, it is vital to perform high-throughput assay to identify TCF7L1-interacting partners, especially those participating in chromatin modification and epigenetic regulation, to explore the mechanisms underlying epigenetic regulation of Keap1 in gastric cancer. Another issue that needs to be addressed is whether TCF7L1 regulates aerobic glycolysis and antioxidant response through or in part through Keap1/NRF2. Answers to this issue might establish a novel TCF7L1/Keap1/NRF2 axis in gastric cancer malignancy regulation.

**Figure 7.**
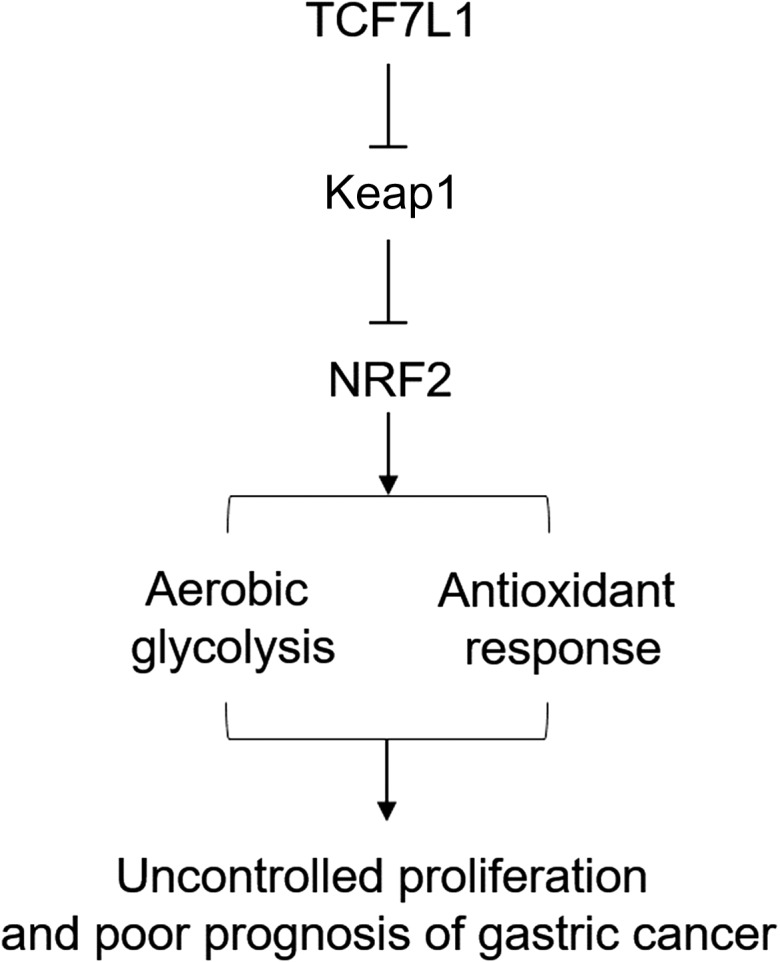
**Schematic representation of the working model** In gastric cancer, TCF7L1 inhibits Keap1 expression, leading to enhanced NRF2 expression and constitutive activation of glycolysis and the antioxidant response, which ultimately contributes to uncontrolled proliferation of gastric cancer cells and poor prognosis in gastric cancer patients.

In conclusion, our present study indicated that TCF7L1 might be a novel marker for the prediction of prognosis of gastric cancer patients. Mechanistic studies demonstrated that TCF7L1 could negatively regulate Keap1 expression and the resultant NRFF2 stability, which positively regulates redox balance and aerobic glycolysis that promote proliferation of gastric cancer cells in patients (**Fig. 7**). The present study provided a novel marker for gastric cancer overall survival, as well as a possible treatment target.
